# Heart rate variability and fatigue in MS: two parallel pathways representing disseminated inflammatory processes?

**DOI:** 10.1007/s10072-022-06385-1

**Published:** 2022-09-20

**Authors:** Guadalupe Garis, Michael Haupts, Thomas Duning, Helmut Hildebrandt

**Affiliations:** 1 Department of Psychology, Carl von Ossietzky University of Oldenburg, Oldenburg, Germany; 2 Department of Neurology, Klinikum Bremen-Ost, Bremen, Germany; 3 Department of Neurology, Heinrich Heine University Düsseldorf, 40225 Düsseldorf, Germany

**Keywords:** Fatigue, Heart rate variability, Multiple sclerosis, Neuroinflammatory reflex, Parasympathetic modulation, Sympathetic modulation, Autonomic nervous system, Vagus nerve

## Abstract

**Background:**

Fatigue is a disabling symptom of multiple sclerosis. Its biological causes are still poorly understood. Several years ago, we proposed that fatigue might be the subjective representation of inflammatory processes. An important step for a straight-forward evaluation of our model would be to show that the level of fatigue is associated with vagal activation. The heart rate is under partial control of the vagus nerve. Using power spectrum analysis allows to separate, at least partly, sympathetic and parasympathetic impact on heart rate variability.

**Methods:**

This narrative review summarizes the evidence for heart rate variability changes in MS patients, their relationship with fatigue and disease course. To do this, we conducted a literature search, including 45 articles relevant to the topic treated in this review.

**Results:**

We illustrate that (1) inflammation leads to a change in cardiac behavior during acute and chronic phases, both in animals and in humans; (2) MS patients show changes of heart rate variability (HRV) that resemble those during acute and chronic inflammation due to multiple causes; (3) existing evidence favors a set of specific predictions about fatigue and parallel HRV changes; and (4) that MS-related brainstem lesions or neurological impairments do not completely explain HRV changes, leaving enough place for an explanatory relation between HRV and fatigue.

**Discussion:**

We discuss the results of this review in relation to our model of fatigue and propose several observational and experimental studies that could be conducted to gain a better insight into whether fatigue and HRV can be interpreted as a common pathway, both reflecting activated autoimmune processes in MS patients.

## Overview

Fatigue is a common and disabling symptom of persons with multiple sclerosis [1]. Patient reports support the existence of distinct components in fatigue [2], some of which are cognitive and others more motor-related. Evidence from previous studies shows that fatigue may be more prevalent amongst patients with progressive forms of the disease [3–5] than those with non-progressive forms of MS. The biological causes of fatigue are still poorly understood. Several years ago, we [6] proposed that one of several origins of fatigue might be the subjective representation of inflammatory processes. According to our model [6], activated T- and B-cells, sessile immune cells, and soluble cytokines cells are detected by the vagal nerve. This information is transmitted to the brainstem and the hypothalamus eliciting a mild form of “sickness behavior.” The signal also reaches the insular cortex and the anterior cingulate, and resulting activity there represents a feeling, i.e., fatigue. The feeling of fatigue acts as an interference during attentional processes that should be directed towards the environment. Consequently, this internally interfering fatigue may affect task performance. However, the degree of interference depends on the task structure: if a task allows mind wandering (like vigilance tasks), the risk of internal interference will be high, if the task does not allow for mind wandering the impact of fatigue will be low. Behavioral impairment may also be high if the neuronal structures, subserving vigilance, suffer from neurodegeneration. This explains why some studies found a correlation between thalamic, basal ganglia and right frontoparietal lesion load and fatigue. However, the impact of lesion load on performance in persons with MS-related fatigue is indirect, i.e., defined by the specific task structure allowing for mind wandering.

One straight-forward evaluation of our model would be to show that patients that experience a high level of fatigue systematically differ from patients with low fatigue regarding their vagal nerve activity. At present, it is impossible to measure such a difference directly, because the vagus nerve system is diffusely organized with small nuclei in the brain stem acting as first relay stations. Moreover, the feeling of fatigue cannot be manipulated experimentally, which would allow us to use a subtraction method necessary for functional brain imaging. In this review about the role of the vagal nerve in fatigue in persons with MS, we will therefore use an indirect measure of its activity. The heart rate is under partial control of the vagus nerve. Using mathematical analyses allows us to separate, at least partially, sympathetic and parasympathetic components of heart rate variability. This review therefore starts with the assumption that an activation of the inflammatory reflex interferes with vagal modulation of heart rate, and this can be measured by an electrocardiogram (ECG). To underpin this assumption, we will first show that inflammation during its acute and chronic phases leads to a change in cardiac behavior, both in animals and in humans not suffering from MS. We then will show that persons with MS show changes of heart rate variability (HRV) that resemble those during acute and chronic inflammation. Having shown that HRV is altered in MS patients, we will discuss whether brainstem lesions, neurological impairment or task load max explain these changes. As a last step, the few studies that already analyzed HRV and the feeling of fatigue in MS patients will be summarized and we will discuss the results of this review in relation to our model of fatigue.

**Fig. 1:**
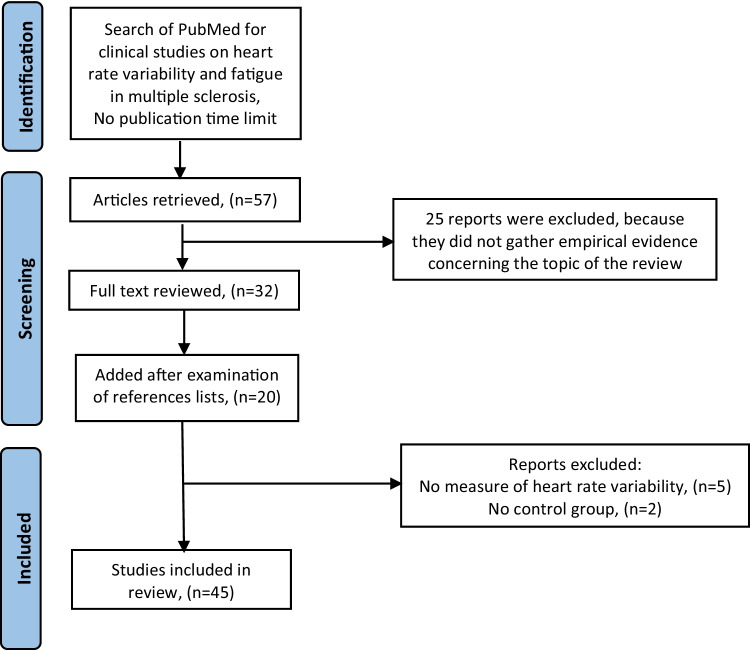
Flowchart of the search strategy

The aim of this narrative review is to sum up current knowledge on fatigue and heart rate variability (in persons with MS) and how the latter may relate to an explanation of fatigue. We have followed the SANRA (Scale for the Assessment of Narrative Review Articles) statement criteria [7] and its explanations and instructions, with a view to improving the quality of this review. A search of PubMed was conducted in August 2021, using the term “multiple sclerosis” in combination with “heart rate variability”, without a publication time limit. Titles, abstracts, and full-text articles written in English were considered for inclusion. The search retrieved 57 articles, of which 32 were selected for review of full text. The other 25 articles were excluded, because they did not gather empirical evidence concerning the topic of the review. A further 20 articles were added from a recent review article on cardiac autonomic dysfunction in MS [8], and from examination of the reference lists of the retrieved articles. Of the remaining 52 articles, 5 were excluded because they did not investigate heart rate variability, and 2 were excluded because they did not include a control group. In total, 45 articles which focused on heart rate variability and perceived fatigue in MS were included in the review (see Fig. 1).

## What is measured by heart rate variability?

HRV is the variation in intervals between consecutive heartbeats [9]. The variation is generated by dynamic changes in the autonomic nervous system (ANS), and it serves as a marker of the combined activity of the sympathetic nervous system (SNS) and the parasympathetic nervous system on heart rate (HR) [10]. It should be noted, however, that the framework to associate the HRV frequency components (LF and HF) with the divisions (PANS and SNS) of the ANS may be restricted, as HRV cannot be completely used as a quantitative measure of the ANS components [11].

A healthy heart does not beat evenly but shows variable and nonlinear oscillations in heartbeats. This variability ensures adaptability to complex and uncertain environments that are in a state of constant change [12].

The ECG of a healthy individual under resting conditions exhibits periodic variation in heartbeats known as respiratory sinus arrhythmia (RSA). RSA refers to the fluctuations of HR according to respiration phases, with HR increasing during inspiration and decreasing during expiration. RSA is predominantly mediated by respiratory gating of parasympathetic efferent activity to the heart. During expiration, vagal nerve efferent trafficking to the sinus node increases, and during inspiration it is attenuated.

HRV can be measured in time- and frequency-domains [10]. Time-domain statistical methods assess the difference between normal R-R intervals (NN), excluding ectopic beats. Time-domain units of measurement include the standard deviation of NN intervals (SDNN), which is the mean of each 5-min segment of a 24-h HRV recording. Another time-domain unit of measurement is the root mean square of the differences in successive R-R intervals (RMSSD), which reflects the beat-to-beat variance in HR and estimates the vagally mediated changes reflected in HRV [13]. In addition, the percentage of normal R-R intervals that differ by 50 ms (pNN50), is closely correlated with parasympathetic nervous system activity (PANS) [13] and the RMSSD. Most researchers, however, prefer the RMSSD to pNN50 [14], because it is more sensitive and less affected by recording duration, HR, or breathing rate.

HRV measures in the frequency-domain encompass high frequency (HF) and low frequency (LF) components. HF power (between 0.15 and 0.40 Hz) relates to RSA and is therefore regarded as parasympathetic in origin. LF power (between 0.04 and 0.15 Hz) is produced by the combined influence of the PANS and SNS and is influenced by baroreflex activity [15], which is mainly vagally mediated [16] LF oscillations are believed to be dominated by sympathetic efferent activity [17, 18]. Nonetheless, whether LF power generally reflects sympathetic cardiac innervation still remains controversial. That is, in resting conditions, the LF band does not reflect sympathetic tone, but baroreflex activity [13, 19–22]. The ratio of LF to HF power (LF/HF ratio) is indicative of SNS to PANS activity and an index of sympathovagal balance. The LF/HF ratio is subject to overt variation depending on activity stress levels. For instance, during rest, the absolute power of LF is less than HF, while during conditions that elicit orthostatic stress, the LF component is higher than HF. HF becomes dominant over LF (thus favoring vagal tone), at very low breathing rates (< 7–8 breaths per minute).

## Vagus nerve, the neuroinflammatory reflex, and heart rate variability

Inflammation can be detected by nociceptive fibers. The autonomous nervous system (ANS) also plays a major role in processing information about inflammation. One essential part of the ANS, which is involved in the immune response, is the vagus nerve. The afferent segment of the vagus nerve expresses receptors for cytokines, but also for tissue damage as a consequence of injuries (usually accompanied by local inflammation). The efferent part of the vagus mediates, among others, the cholinergic anti-inflammatory pathway, by activating immune cells in the spleen and by synapsing its fibers onto neurons that release acetylcholine (Ach, primarily alpha-7 nicotinic receptor units [23]) at the synaptic junction with macrophages [24]. When ACh binds to alpha-7 nitotinic ACh receptors of those macrophages, it suppresses the release of tumor necrosis factor, a pro-inflammatory cytokine [23].

To construct a balance between inflammation as a destructive response to immune challenges and inflammation as a necessary repair process, the vagus nerve shifts local processes into the direction of the latter [25]. The efferent action of the parasympathetic system during the response to inflammation is under control of different brainstem (e.g., the dorsal nucleus of the vagus), midbrain (e.g., the freezing center of the periaqueductal grey matter), and cortical centers (e.g., the insular cortex) [25, 26]. Afferent projections of the vagus project to the solitary nucleus (SN) in the medulla oblongata. There is a topological representation of different parts of the body in the SN. Moreover, the SN is interconnected with various other nuclei of the brainstem and higher brain areas, integrating responses from other parts of the ANS, but also motor responses of the organism. In the case of inflammation, in addition to the immediate peripheral and local response there is a more global response initiated by the periaqueductal grey matter and the hypothalamus. Higher order cortical structures can nevertheless diminish the midbrain response to inflammation. This integrated behavioral response has been termed “sickness behavior” with symptoms like fever, fatigue, extended periods of sleep, loss of appetite, and motivation. Accordingly, a study by Eisenberger et al. [27] found increased reported depressed mood that resembled “sickness behavior” among participants who were exposed to an endotoxin which significantly raised interleukin 6 (IL-6) levels, and this effect correlated with greater fMRI activity in the dorsal anterior cingulate cortex and in the right anterior insula. In line with the former, increased fMRI activation of interoceptive brain regions (i.e., anterior cingulate cortex and insula) has been also observed by Harrison et al. [28] after typhoid vaccination of healthy participants induced a robust inflammatory response by increased circulating IL-6, simultaneously generating symptoms of sickness, such as fatigue, confusion, and impaired concentration. The later findings show that inflammatory activation implicates central nervous system function to elicit core symptoms of sickness behavior.

In this case, the representation of higher order goals of the organism leads to a top-down control of sickness behavior. Smith et al. [24] examined the hierarchical basis of neurovisceral integration arguing that the interaction and interplay of increasing total organismic response to challenges of the ANS is regulated by different set points (“priors”) and that long-term control of lower responses or the experience of traumatic deviations from expectations may shift such priors chronically to different states, which may be detrimental in the long run.

The vagal nerve is not only involved in the neuroinflammatory reflex, but also in many other actions of the PANS: One prominent aspect is the regulation of the heart rate. Stimulation of the right vagus nerve leads to bradykinesia, whereas stimulation of the left vagus nerve does not imply a slowing of heart rate [24]. In general, short-term changes in heart rate are triggered by the vagus nerve, whereas long-term changes (encompassing several minutes or hours) are afforded by the sympathetic nervous system (SNS). The baroreflex, for example, responsible for short-term blood pressure regulation, is under vagal control during upright standing (“orthostasis”). The central origin of the baroreflex is located in the ventrolateral superficial reticular formation of the brain stem and initiates additional activity in the SN and dorsal nucleus of the vagus as additional control centers [25]. The SN also receives excitatory stimulation from the Bötzinger complex, which is involved in the modulation of respiratory depth and rhythmicity. Deep breathing is part of the assessment of the cardiovagal reflex and results in increased short-term heart rate variability because of PANS dominance. Thus, there is a cascade of ascending brain stem centers integrating PANS and SNS control of an organism’s response to external affordances and internal goals for action.

## Heart rate variability and inflammation

### ANS disorder and multiple sclerosis

Our model proposes that fatigue is a subjective representation of inflammatory processes due to either activated T-cells that occasionally pass the blood brain barrier initiating immune processes against the myelin sheath of axons, or intrathecally trapped B-lymphocytes and sessile immune cells [29, 30]. One prediction that can be derived from our model is that stimulation of the vagus by inflammatory processes might also lead to changes in other autonomous functions. Many epidemiological studies demonstrated a similar intercorrelation of ANS-related symptoms in MS patients. Investigations that used the COMPASS-31 questionnaire, focusing on ANS dysfunctions, have shown that MS patients suffer from more ANS-related symptoms than healthy controls (especially for pupillomotor symptoms and orthostatic intolerance), and that these symptoms correlate with fatigue [31–34], whereas no relationship between COMPASS-31 and EDSS, or disease duration could be established by Cortez et al. [35].

The vagal nerve is involved in digestion and bladder control. Lin et al. [36] found that fatigue, intestinal symptoms, or deficits in bladder control are correlated. A large study in the United Kingdom showed that MS patients made more visits to health practitioners in the years and sometimes decades before their diagnosis compared to the general population. Moreover, it appeared that ANS dysfunctions (including fatigue) played a major role in these visits and that ANS dysfunctions experienced early in the disease became more severe during the first two years after the diagnosis [37, 38]. In another study, Almeida et al. [39] were able to demonstrate that bowel symptoms preceded the clinical isolated syndrome by about 3 to 4 years, and they correlated with retrospectively scored fatigue.

### HRV changes during acute inflammation

Subjective symptoms assessed with questionnaires are always prone to reporting bias, even when statistically controlled for. In the following, we will show that inflammatory processes alter HRV, an objective measure of ANS activity.

Infusion of lipopolysaccharides (LPS) leads to an immediate response of the immune system, and therefore it has often been used to study the biological and behavioral consequences of inflammation. Several animal studies demonstrated that shortly after LPS infusion, levels of cytokines, such as IL-6 or TNF-α etc., increased, but also changes in heart rate were observed. A few human studies have also used this design. Jan et al. [16] challenged 30 young participants with 2 ng/kg endotoxin. They studied the bodily responses with serial blood probes and by monitoring heart rate. About one hour after the challenge TNF, IL-6, IL-8, an IL-10 started to increase steeply. There was a parallel shar*p drop* of SDANN, pNN50 and HF power of heart rate. The LF/HF ratio also changed but in a less steep manner. After 24 h, the scores normalized. Herlitz et al. [40] repeated this study with different dosages of endotoxin to analyze the immune response at different levels. Changes in body temperature were the most sensitive marker, present already after a challenge with 0.25 ng/kg. Changes in TNF-alpha and IL-6 together with an increase of heart rate were observed as a response to 0.5 ng/kg. HRV changes differed significantly from placebo starting with doses of 1 and 2 ng/kg. These and other studies [41, 42] argue in favor of a common and orchestrated PANS response to inflammatory challenges. The latter results provide evidence that circulating inflammatory markers, argued to be involved in the development of sickness behavior, drop HRV parameters of PANS activity, suggesting that inflammation may indeed contribute to the pathogenesis of MS-fatigue as an expression of resulting sickness behavior.

### HRV changes in chronic inflammatory diseases

Is the vagal response restricted to an acute phase or still present during chronic inflammation? Reviews examining HRV changes in chronic (auto-)immune diseases argue for a lasting change in vagal activation. Haensel et al. [43] reviewed thirteen studies on inflammatory markers in cardiovascular disease and HRV. Most studies focused on IL-6 and C-reactive protein (CRP) and found a significant negative correlation with the HRV parameter SDNN, which is most frequently for assessment. Not only SDNN and HF decreased in the patient groups but also other parameters indicative of sympathetic modulation of HRV. Haensel et al. [43] concluded that, while inflammatory markers appear to be related to HRV, this concerns parameters reflecting both parasympathetic and sympathetic activity.

Kim et al. [44] reviewed the relation between HRV and inflammatory bowel disease. They were able to include 10 studies on this topic with 440 patients and 208 healthy controls. HF, SDNN, and pNN50 measures were lower in the patient groups. Moderate to high effect sizes were present for all HRV measures except for LF. They concluded that inflammatory bowel disease is strongly associated with restricted HRV and that PANS-related HRV seems to be more closely related to chronic inflammation compared to SNS-related HRV.

Provan et al. [45] reviewed the relation between HRV and rheumatoid arthritis. They were able to include 21 papers in their review. Interestingly, they also analyzed the published results on cytokines and HRV. Their results suggested that inflammation, present at the time of the HRV measurement and measured by the concentration of cytokines in the blood, plays a greater role in determining HRV than disease duration or cumulative disease activity. Related to HRV, RMSSD and HF were used in several studies allowing for a calculation of the effect size, and both were decreased compared to healthy control. Thus, persons with rheumatoid arthritis appear to show reduced PANS-related HRV.

Crosswell et al. [46] analyzed whether fatigue in breast cancer survivors is related to HRV, CRP and IL-6 levels. They included 84 women and focused on resting HRV (no physical or mental load during the examination). All participants had suffered from cancer before reaching the age of 50. High fatigue correlated with a lower HRV and increased concentrations of IL-6. There was also a correlation between IL-6 and RMSSD or HF. After controlling for age, physical activity, and body mass index, the correlation between HF and IL-6 remained significant as well as the correlation between fatigue and RMSSD. Crosswell et al. [46] concluded that research on the etiology of cancer-related fatigue should give attention to ANS dysfunctions.

Williams et al. [47] reviewed 51 studies (irrespective of etiology) on the relation between inflammatory markers like CRP, IL-1, IL-6, or TNF and HRV. They found that SDNN and HF showed the strongest negative associations with HRV. SDNN correlated with CRP, IL-6, and WBC, whereas HF correlated with CRP, IL-1, IL-6, IL-10, TNF, WBC, and fibrinogen. All these correlations were negative, i.e., low PANS-related HRV was associated with high counts of proinflammatory cytokines and vice versa.

### HRV and fatigue in MS patients: defining some characteristics derived from the phenomenology of fatigue and structural assumptions of our model

Summarizing the results of the experimental studies and those in chronic inflammatory diseases, we conclude that there is combined evidence that inflammatory processes not only elicit a neuroinflammatory reflex but also decrease PANS-related HRV and trigger the feeling of fatigue. However, the mere presence of fatigue and PANS-related changes of HRV is only a weak argument for a causal interrelation. In the following section, we will specify features of fatigue in MS (based on Sander et al. [48]), which allow a formulation of more specific predictions.

One major feature of MS-related fatigue is that it is only very loosely related to the level of mental or physical load. Fatigue can arise even after minimal effort or in the absence of effort. It can develop instantly and is unpredictable for a patient. Thus, according to these defining characteristics that separate MS-related fatigue from increased tiredness, it can be expected that HRV changes, if they are related to fatigue, are already present during rest and not only after any kind of effort.

This assumption can be stressed further: inflammatory processes do not stop during sleep, a status without any environmental load. Our model assumes that the HRV changes are present during sleep, probably even more so than during the day, because during the night there is a circadian-mediated increase of inflammatory processes.

According to our model, fatigue is not related to neurological impairment. If the biological basis of fatigue is the neuroinflammatory reflex, changes in HRV depend on the detection of cytokines by the vagus in the periphery. The expression of cytokines can be high (even if the activated T-cells do not pass the blood brain barrier) or low during different phases of the disease course. However, a link between HRV and ongoing disease activity in MS (as reflected by EDSS) has been suggested [49] (being just another part of accumulating neurological deficits). Thus, our model predicts a constant *inflammation*-based association between fatigue and PANS-related HRV (see the “HRV changes during acute inflammation” and “HRV changes in chronic inflammatory diseases” sections of this review). However, there might also exist an increasing *accumulation*-based association between disease duration and HRV.

As a correlation of this latter prediction, we assume that PANS-related HRV changes should be common (like fatigue), but should not arise due to brain lesions. This does not exclude that ANS dysfunctions related to brain lesions may be present in MS patients (due to brain stem lesions, for example)—however they should affect only a minority of the patients (with increasing frequency related to a higher duration of the disease).

## Changes of HRV in multiple sclerosis

### HRV adaptation in rest and during orthostatic challenge

Numerous studies have provided evidence of autonomic dysfunction involving the cardiovascular system of MS patients (reviewed in depth by Findling et al. [8]). The most prevalent finding is a lower overall parasympathetic output in relapse-remitting MS (RRMS) patients in comparison to healthy controls (HC), as demonstrated by significant reductions in long-term monitoring (24-h electrocardiogram) [50–53] and short-term HRV analysis [54]. In studies that measured the strength of each HRV frequency component at rest (while sitting or in supine position), variant-undefined MS patients were more predisposed to reduced parasympathetic activity at rest than HC [54–58]. However, Reynders et al. [49] found that even though the parasympathetic parameters SDNN and RMSSD showed a strong correlation at baseline and after 3 months within the MS group, they showed little intra-individual reproducibility.

Two studies also analyzed if the differences between HC and MS patients increase or decrease during sleep. Both studies found that the ANS associated difference in cardiac activity increases during the night [59, 60].

Different patterns of HRV have been observed during and outside supine resting conditions. Baroreflex activity, as reflected by the LF power component of HRV in resting conditions, was found to be lower in RRMS [50, 51] and in MS variant-undefined patients [52, 55, 61] in relation to HC. In the study by Monge-Argilés et al. [60], variant-undefined MS patients had a lower sympathovagal tone during the supine resting condition than HC, while sympathovagal tone increased outside of the rest condition of the 24 h recording. The divergent results in sympathetic tone between the rest condition and the non-rest part of the examination could be explained by more experience (and then better relaxation) for clinical examinations in MS patients, than in HC.

Expected changes in ANS, as reflected by HRV variations in response to orthostatic stress conditions (sit-to-stand transition and head-up tilt tests) and physical exercise, have been tested on MS patients in comparison to HC. During an orthostatic challenge and activity, healthy organisms typically experience an augmented sympathetic tone, along with inhibition of the cardiovagal system, in order to sustain the necessary increase in HR. Accordingly, expected HRV variations during head-up tilt test (sympathetic predomination and lower vagal tone) have been reported in the variant-undefined MS group [61]. In contrast, Rzepinski et al. [62] established that RRMS patients are more predisposed to a combined deficit in sympathetic and parasympathetic responses to the head-up tilt test, compared to HC. Moreover, Gervasoni et al. [57] found a lower RMSSD in variant-undefined MS patients than HC post-exercise recovery, which suggests that inhibition of the vagal tone may remain preserved in MS. On the other hand, others did not observe any changes in HRV variables between variant-undefined MS patients and HC after orthostatic challenge [63, 64].

Targeted changes in ANS activity are also known to occur in response to deep breathing and relaxation [65]. That is, deep breathing techniques increase vagal nerve activity, signaling the PANS to calm the body down [66] due to an entrainment between respiratory rate and vagal tone [67]. Abnormalities in vagal power in MS have been detected at slower respiratory rates during paced breathing exercises, as reflected by lower HF power in the variant-undefined MS group than HC, at 8, 12 and 15 breaths per minute (b.p.m), but not at 18 b.p.m [56]. Further, a recent review demonstrated that yoga practices, which heavily rely on deep breathing exercises, may be particularly helpful in alleviating MS-related fatigue [68], arguing for a temporary increase in PANS activity. Judging from this evidence, increasing HRV through deep breathing exercises could presumably produce body autonomic activity characteristic of relaxation in MS patients, helping to restore parasympathetic balance.

Summarizing this section, there is clear evidence of a PANS-related HRV change during rest and sleep, unclear evidence or a lack of sympathetic modulation during orthostatic challenge and activity, and weak evidence of decrease vagal response to deep breathing in MS patients.

### Relation to disease activity and risk of relapse

It has already been mentioned in the “ANS disorder and multiple sclerosis” section that epidemiological studies argue that alteration of autonomic functions may define something like an MS prodrome [37, 38]; these already being present years before initial inflammatory processes give rise to clinically relevant impairments. Focusing on the experience of a relapse, different patterns of HRV have been associated with an increased risk of MS disease activity [63] and HRV changes may happen in response to elevated inflammation during the prodromal phase of a MS relapse. In active RRMS patients, relapse rate, even sub-clinically, was associated with lower sympathetic tone, whereas stable RRMS patients did not differ from HC [63]. It was also observed that patients with incomplete recovery from relapse showed less sympathetic increase after head-up tilt test during the relapse, compared to subjects who completely recovered. Notably, the severity of relapse, assessed by an EDSS increase during the relapse, did not correlate with the HRV parameters [63]. These results suggest that during the relapse, the sympathetic system is most affected with a relative sparing of the parasympathetic system.

Reynders et al. [49] found that higher HRV parasympathetic parameters predicted MS relapse after 3 and 6 months. Nonetheless, they used self-reported relapses, which were not necessarily confirmed by a physician. Another study contradicted the previous results by reporting that parasympathetic HRV parameters were not significantly related to relapse rate [53]. In line with a presumed association between neuronal inflammation and HRV, Gidron et al. [69] found a negative correlation between parasympathetic tone and neurofilament light chain, an identified biomarker of disease activity in MS [70] in variant-undefined MS patients without relapse.

Due to these contradicting findings no definite conclusions can be drawn about HRV directly before and during an acute relapse. Given the modern immunohistological perspective of ongoing neuroinflammatory processes even in MS appearing clinically “stable,” as well as early degenerative changes in persons with MS classified “relapsing–remitting,” assorting conventional clinical MS classifications may not help for further understanding [30, 71].

### Relation to lesions of the central nervous system

Autonomic abnormalities in MS are usually explained by widespread demyelinating lesions in areas of the brain that exert influence on cardiovascular reflexes and autonomic regulation [72]. Specifically, cardiovascular autonomic disruption may happen due to plaques distributed throughout the brainstem and spinal cord affecting autonomic regulatory areas and their connections [55, 73–75].

Demyelination is likely to interfere with the descending ANS brainstem pathways [63, 73]. It is therefore reasonable to examine a possible association between a HRV impairment and the presence and localization of CNS lesions in MS patients.

The relationship between brain and spinal lesions as detected by magnetic resonance imaging (MRI) and HRV has been researched in studies with stratified patients according to the presence or absence of demyelinating lesions, mainly involving the brainstem and cervical spinal cord. While the presence of brainstem lesions has been linked to lower parasympathetic tone [76], spinal lesions have been associated with a higher sympathetic tone [54], and insular lesions correlated with sympathovagal predominance [54]. However, Videira et al. [54] retrospectively analyzed white matter lesions near these CNS structures and did not specify where they were situated. In contrast, a recent study that investigated PANS-related HRV found no significant differences between MS patients with and without brainstem lesions [51]. Likewise, a study from the 1990s revealed no significant correlations between the total lesion area in both hemispheres or their specific localization and any of the frequency-domain HRV frequency parameters [61]. A similar finding was reported by Damla et al. [50] who found no significant relationship between brainstem, cervical and thoracic spinal lesions and time- and frequency-domain HRV analyses. Similarly, the presence of demyelinating lesions in the brainstem was not attributed to significant differences in the sympathovagal ratio [63].

We conclude that there is no clear evidence to assume an association between HRV impairment and structural damage to the brain in MS.

### HRV, disease duration, and functional impairments

Several investigators directly compared HRV differences in MS patients in relation to the *duration of the illness*. First, early involvement of ANS dysfunction in MS has been validated, with PANS-related HRV changes to be present already in newly diagnosed RRMS patients [49]. The duration of MS illness seems to provoke more pronounced parasympathetic deterioration, as confirmed by several investigators [52, 61, 63]. Progression of the disease was reported to correlate with increased parasympathetic dysfunction [77]. Others, however, did not observe changes in HRV parameters in prolonged MS [54, 56, 76–79]. A study including RRMS patients, determined that those with more than 5 years since formal diagnosis had a stronger decrease in PANS-related HRV than those with less than 5 years from diagnosis [52]. This is in line with the study by Studer et al. [63], who showed that more than 5 years of MS disease duration correlated with a lower parasympathetic tone.

Overall, findings indicate that the reduction in parasympathetic tone amplifies over time in MS. That is, the longer the disease goes on for, the more profound the parasympathetic dysfunction.

However, sympathetic dysfunction can also be present very early as evidenced in patients with clinically isolated syndrome [76]. As the disease progresses over time, sympathetic activity becomes prevalent [57, 63], whereas expected sympathetic reactivity to orthostatic stresses weakens [55]. Others, however, did not observe a significant relationship between sympathetic reactivity to orthostatic stressors in MS and disease duration [52, 64, 76, 78]. In conclusion, sympathetic activity may be weakly reduced already throughout the early phases, but then it chronically evolves into sympathetic predominance, weaker sympathetic reactivity, and parasympathetic deficiency.

HRV impairment has also been connected to *disease-related functional impairments*. In MS, lower PANS activity [60, 79], and SNS hyperactivity [63] are more apparent with increasing severity of functional impairments, as measured by the Expanded Disability Status Scale (EDSS). Accordingly, less affected MS patients are found to preserve higher levels of HRV [60]. Furthermore, Studer et al. [62] revealed that SNS hyperactivity was more manifested among severely disabled PPMS patients. In the study by Studer et al. [63], disease severity was associated with increased disruption of sympathetic reactivity during head-up tilt test in PPMS patients, while no difference was found between RRMS patients and HC. Likewise, in the study by Zawadka-Kunikowska et al. [79], a higher overall EDSS score was linked to a higher sympathovagal ratio at rest and lower parasympathetic parameters at rest in variant-undefined MS patients. Other investigators did not find a similar significant difference [35, 53, 54, 61]. Overall, functional deterioration leads to a decrease in parasympathetic activity, but maintenance of an increased sympathetic tone compared to HC. However, the increase sympathetic tone was found in PPMS patients, and we will discuss the possible course of the disease and HRV changes below.

It should be noted that in some of the studies described above [58, 61, 79], patients took an immunomodulating medication “Fingolimod” as a treatment for RRMS. Fingolimod and various other immunomodulating medications have been found to diminish cardiovagal responses to autonomic challenges as well as to reduce cardiac autonomic modulation at rest [8]. Moreover, age must be considered as a confounding factor discussing disease duration, related functional deterioration and MS-related HRV changes. In the study by Gökaslan et al. [51], all patients had been diagnosed with MS within the last 3 years, and parasympathetic HRV parameters significantly decreased as patient age increased. This is in line with McDougall and McLeod [72], who showed that HRV parameters decreased as persons with MS aged. These findings argue that parasympathetic activity decreases, and sympathetic activity becomes more dominant as patients age, regardless of disease duration.

### HRV in different MS disease courses

A difference in patterns of dysautonomia among MS variants has been established. Compared to HC and patients with RRMS, patients with PPMS have been particularly associated with higher sympathetic predominance at rest [61, 63, 79], and lower sympathetic reactivity during head up tilt-up [63, 79, 80] even when corrected for age, sex, and disease duration [80]. Specifically, a heightened basal sympathetic tone in PPMS, related to inadequate sympathetic reactivity during orthostatic stress [7]. However, in the study by Gervasoni et al. [57] sympathovagal parameters during orthostatic challenge were similar in the PPMS and RRMS groups, and in the PPMS and HC groups. Also, Zawadka-Kunikowska et al. [79] observed impaired vagal inhibition after a head-up tilt test in the progressive MS group, comprised by 17 secondary progressive (SPMS) patients and 4 PPMS patients, indicating insufficient withdrawal of PANS modulation after orthostatic challenge. Thus, PPMS subjects cannot adapt during orthostatic challenge, and therefore do not reach expected sympathetic increase and parasympathetic inhibition. Given the set of associations described above, the PPMS variant involves a stronger impairment in physiological sympathetic reactivity, along with a shift of the sympathovagal modulation toward sympathetic predominance, as compared to the RRMS and HC.

PPMS patients are often diagnosed at an older age than RRMS patients, which may play a confounding role for the interpretation of results. Still, the vast majority of studies reviewed here matched for age [52, 54–58, 60–62, 64, 66, 68, 81], most demonstrating that cardiac parasympathetic dysfunction is a common denominator in MS. Thus, MS disease courses have been found to influence the ANS differently.

In sum, autonomic balance is substantially altered in the MS patient group compared to HC, and the alterations are more apparent in the primary progressive variant of MS. Indeed, patients with PPMS were found to have lower parasympathetic activity and higher sympathetic tone at rest and lower expected sympathetic reactivity in response to orthostatic challenges. For RRMS patients only a lower parasympathetic tone has been documented, with scores between HC and PPMS patients. An increased basal sympathetic tone was associated with the failure of sympathetic reactivity, and this is a further index of autonomic impairment in the PPMS variant, suggesting that the SNS of PPMS patients is overactive and exhausted.

It should be mentioned that a lack of reactivity of the SNS in PPMS could be associated with the limitation of physical activity and resulting cardiovascular deconditioning in severely disabled PPMS patients. For instance, Goldsmith et al. [82] found that untrained participants had a lower LF power than trained ones. Because of that, comparing hypoactive MS patients and normally active subjects could prove, in relation to the LF parameter, a difference that has been reported between endurance-trained and untrained individuals.

## What is known about HRV and fatigue in MS

Three studies addressed the topic of fatigue and HRV indirectly. Cleland et al. [83] analyzed the role of physical activity on bone mineral density in 23 persons with MS compared to 22 control participants. They also assessed fatigue, depression, cytokines and HRV (only SDNN) in both groups, however evaluated only their relation to bone mineral content and not their interrelations. MS patients did not differ from healthy controls in HRV, but in IL-6 and TNF-alpha concentrations. Unfortunately, there is no description of how HRV was measured (supine, during sitting or standing) and if it correlated with fatigue. Therefore, these results are difficult to interpret.

Rampichini et al. [84] studied the relation between HRV and sub-maximal physical exercise in 17 persons with MS and 17 healthy controls. They also measured fatigue. However, the fatigue measure was restricted to physical and muscle exhaustion after the sub-maximal workout, it did not concern fatigue as measured with validated questionnaires and did not fit the definition of fatigue as being *not* explained by prior external effort. Rampichini et al. [84] included older patients (average age of 56 years), being already impaired in walking (EDSS of 5.9) and suffering from different disease courses. RMSSD, the only HRV parameter they analyzed, was decreased in patients before the exercise and remained decreased afterwards. The difference between the two groups did not change during exercising. HRV did not correlate with fatigue measures before and after the physical exercise. The significance of this study for this review is unclear because it focused on (muscle) exhaustion not on fatigue.

Increased heat sensitivity exaggerating fatigue is a common symptom in MS and there are several reports that cooling the body leads to a reduced feeling of fatigue [85]. Meyer-Heim et al. [84] studied the impact of advanced lightweight cooling-garment technology on fatigue and HRV in MS. Cooling influenced motor, but not cognitive performance of the MS patients. In the subgroup of 6 patients with perfect ECG data, the HRV was reduced in the sham condition, whereas it approached the level of healthy controls during cooling.

Gossmann et al. [86] studied the effect of cooling on fatigue in 29 persons with MS and 10 healthy controls using a 20-min-long vigilance task to induce fatigue and they measured HRV (focusing exclusively on the LF/HF ratio) during the assessment. Only three of the MS patients did not suffer from fatigue and all relevant statistical evaluations were repeated after their exclusion. MS patients produced more omissions during the vigilance task and their number of omissions increased significantly during the task. Concerning the LF/HF ratio, the difference between the persons with MS-related fatigue and the healthy controls approached significance. MS patients showed a lower LF/HF ratio than healthy controls. Moreover, during the vigilance task the LF/HF ratio increased in the control group but did not show any change in the MS patient group. However, wearing the cooling vest had no measurable effect on body temperature, and had no impact on HRV and fatigue. As a result, it can be argued that compared to healthy controls MS patients with fatigue show a lower LF/HR ratio and do not respond with heart rate variability change if challenged by a vigilance task.

In one of the first studies using HRV analysis related to fatigue in MS patients, Keselbrener et al. [81] included just 10 MS patients with a Fatigue Severity Scale (FSS) score above 3.5 (which is below the cutoff score of 4 for fatigue in the FSS). They also used frequency ranges for calculating VLF, LF, and HF, which are uncommon today: 0.02–0.06, 0.06–0.2, and 0.2–0.5, respectively, instead of VLF: 0.0033–0.04 HZ, LF: 0.04–0.15 HZ, HF: 0.15–0.4 HZ. Compared to healthy controls, only the LF/HF ratio approached significance. Further analyses showed that MS patients showed a reduction of vagal activity related to age, whereas healthy controls did not. However, the small number of patients do not really allow for subgroup analyses.

Merico [87] analyzed different ANS parameter in 22 MS patients in supine position, during deep breathing and during standing. Unfortunately, the paper does not offer information about the results in different HRV parameters. However, Merico [87] reported that stress did not induce an increase in autonomic variation (as expected in healthy subjects) and that this concerned mostly a loss of parasympathetic modulation. Moreover, fatigue measured by the FSS correlated with autonomic parameters, but the average score of experienced fatigue was not reported.

Yu et al. [88] analyzed HRV in 17 MS patients with fatigue and 9 healthy controls. The fatigue score was moderate to severe with an average of about 6 in the FSS. They found that PANS-related HRV was reduced in the MS patients in most of the assessed conditions. Because they did not include a group of MS patients not suffering from fatigue, it is unclear whether the difference is due to the disease or to the feeling of fatigue and no specific comparison between rest and activity periods were performed. However, this study (and [86]) is one of the very few looking for the sequelae of a cognitive task on HRV. They used a learning task, presenting 20 figures that the participants had to recall subsequently. They also found that during this task without any specific body movements the HRV parameters of LF and HF differed between the patients and the controls.

Sander et al. [65] treated 50 persons with MS-related fatigue with a single trial containing two different kinds of a biofeedback intervention. They looked for the impact of the two biofeedback exercises on fatigue before and after performing a vigilance task. The MS patients were divided by those suffering from severe fatigue (defined by the total score of the FSMC) or from weak to moderate fatigue. Sander et al. [65] focused their HRV analysis on SDNN and pNN50. They found that the group with high fatigue showed significantly lower SDNN and pNN50 scores than the group with weak to moderate fatigue. Moreover, they found that progressive muscle relaxation (one of the two biofeedback interventions) led to an increase of the SDNN and the pNN50 scores, but only for the group with weak to moderate fatigue. The group with severe fatigue did not respond with any relevant changes in the two HRV scores.

In a former study, Sander et al. [89] analyzed which HRV parameter (VLF, LF, HF, SDNN, RMSSD, pNN50) predicted total fatigue, as measured with FSMC. Regression analysis showed that VLF and HF were the best predictors. In sum, these two studies found that the total score of the FSMC is related to VLF, two parameters primarily indicating short-term fluctuation, which are closely related to vagal activity. SDNN and pNN50 are sensitive to biofeedback induced PANS activation, but MS patients with high fatigue are not able to learn to induce such changes. Similarly, Merico [87], Yu et al. [88], and (in interaction with age) Keselbrener et al. [81] found primarily PANS-related HRV reduction related to fatigue, measure with the FSS.

## HRV and fatigue in MS: a common pathway of disseminated inflammatory processes?

The present review highlights the possibility that in MS, activated T- and B-cells, sessile immune cells, and soluble cytokines induce ANS activity, and this is reflected in HRV parameters. We also proposed that fatigue is associated with an altered HRV, because, according to our model 1, HRV and fatigue share a common neural substrate, i.e., vagal processing. To analyze this assumption, we developed a set of criteria for defining fatigue in  “HRV and fatigue in MS patients: defining some characteristics derived from the phenomenology of fatigue and structural assumptions of our model” and we predicted alterations in HRV.

Fatigue can be regarded as a feeling arising independently from environmental challenges, that can subsequently be experienced during rest. Thus, we hypothesized that changes in ANS that are reflected in HRV should be present during rest. We found that, irrespective of disease duration and disease course, MS patients demonstrate a lower PANS-related HRV during rest and during deep breathing (“HRV adaptation in rest and during orthostatic challenge” section). Studies directly measuring the relationship between HRV and fatigue also demonstrated that MS patients with fatigue have a lower PANS-related HRV and that the LF/HF ratio correlated with fatigue (“What is known about HRV and fatigue in MS” section) [65, 81, 87]. A study by Sander et al. [65] compared MS groups with differing fatigue status on HRV parameters and implemented an experimental intervention (biofeedback). Persons with MS with *severe* fatigue showed no expected increases in HRV levels in response to the biofeedback intervention. Those with *mild to moderate* fatigue responded with PANS-related HRV changes to the relaxation condition and did not show differences in SNS-related HRV. This would suggest that beyond the severe fatigue threshold, MS patients do not react in the optimal ranges to changing internal conditions.

Inflammatory processes do not cease during sleep. Cortisol production drops to its lowest point around midnight, resulting in increased inflammation. Because of this, we expect HRV changes to become more pronounced during sleep. Accordingly, Monge-Argilés et al. [60] and Ferini-Strambi et al. [78] found that during the night, parameter differences between MS patients and the control group seemed larger. Moreover, 24-h measurements taken at an early stage of the disease revealed increased levels of PANS-related HRV activity, whereas those taken at a later stage of the disease revealed decreased PANS-related HRV activity [55–57, 78].

If the biological basis of fatigue is the neuro-inflammatory reflex, then afferent vagus nerve signaling, activated by cytokines in the body, would be accompanied by changes in HRV. Cytokine expression can be high (even if activated T-cells do not pass the blood–brain barrier) or low at different phases of the disease course. Hence, fatigue feelings should not be closely related to disease duration. The results of the present review confirmed this prediction (“HRV, disease duration, and functional impairments” section). We found that RRMS patients have lower PANS-related HRV activity than HCs. The lower HRV was derived from time domain parameters of HRV (SDNN and pNN50), and frequency domain parameters (HF). The association between levels of inflammatory biomarkers (interferon γ and tumor necrosis factor α) and MS-fatigue [90, 91] may have limited relevance to progressive forms of MS due to the absence of a marked inflammatory response [92, 93]. One study argued that RRMS patients suffering an acute relapse may have increased PANS-related HRV activity relative to SNS-related HRV activity. However, the latter finding has not been replicated in other studies (“Relation to disease activity and risk of relapse” section). As shown in the “HRV changes during acute inflammation” and “HRV changes in chronic inflammatory diseases” sections, such an increase in PANS-related HRV would contradict the findings from other acute and chronic diseases. Therefore, the available evidence argues equivocally that PANS-related HRV *decrease* during ongoing inflammatory processes.

According to our model, focal brain atrophy may produce similar effects on behavioral performance due to the interfering feeling of fatigue, but brain atrophy does not lead to fatigue. Thus, HRV may change in parallel with EDSS (reflecting accumulated neurological deficits) and with increasing brain lesions, however, their statistical correlation would be low. Our review revealed that both assumptions seem to be correct: (1) there was no conclusive evidence for a correlation between the EDSS score and HRV (“HRV, disease duration, and functional impairments” section), (2) there was no conclusive evidence for an association between brain damage and HRV changes either (“HRV in different MS disease courses” section).

Another result of our review is that, in parallel with the disease course, PANS-related HRV decreases even further. First, some studies showed age-related changes in PANS-related HRV in MS patients but not in HCs. Second, a reduction in PANS-related HRV was found more often in chronic MS patients than in RRMS patients. However, at the same time, duration of disease and disease course also led to increases in HRV indices that reflect SNS activity. Sympathetic dominance is already present during rest and is not influenced by orthostatic stress (no significant results for the head-up tilt test). Therefore, in the later phases of the disease, a more pronounced imbalance between PANS and ANS is present. It should be noted that our model does not predict any relation between SNS activity and fatigue, because the SNS seems to be unaffected by inflammatory processes in the body. Consequently, our model does not predict that a higher imbalance between PANS and ANS would lead to higher levels of fatigue nor that the disease duration or the conversion to a chronic phase would be associated with higher levels of fatigue.

In summary, it can be stated that MS patients differ from HCs by having less activity levels of PANS-related HRV and that these are inversely correlated with fatigue. At first sight, this seems to contradict our assumption, because a high state of parasympathetic activity should be related to high HRV, a causal relation used, for example, to assess the cardiovagal reflex. However, after reviewing experimental animal studies on HRV in acute and chronic human inflammatory diseases (“HRV changes during acute inflammation” and “HRV changes in chronic inflammatory diseases” sections), we were able to show that an increased level of pro-inflammatory cytokines and activated T-cells, which induces vagal processing, is related to *lower* PANS-related total HRV even with higher parasympathetic activity. Therefore, analyses of acute and chronic inflammatory processes in various diseases fit the results published of MS patients, with a prominent decrease in PANS-related HRV, arguing for a relation between inflammation and PANS-related HRV, however, in the opposite direction as it may be expected.

**Fig. 2:**
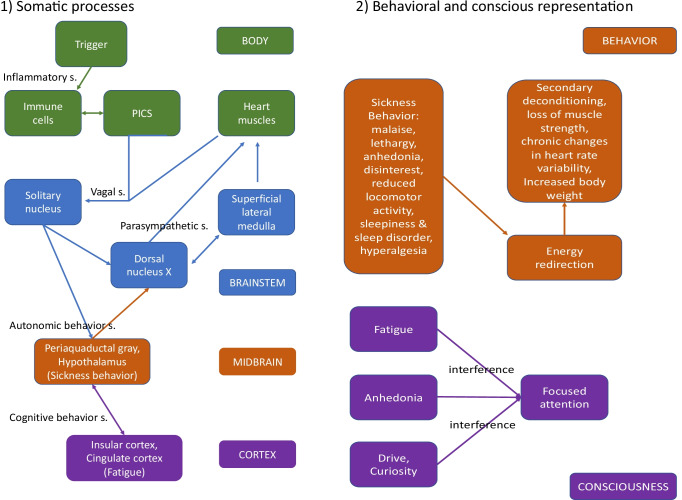
Schematic drawing of our model. (1) Somatic processes. (2) Behavioral and conscious representation. s. = setpoint. *Somatic processes (left side)*: The existence of casual triggers leads to the activation of autoimmune responses. Previous exposure to these triggers might have led to a changed of the “prior,” the set-point for starting and regulating the autoimmune response. The production of pro-inflammatory cytokines leads to the activation of the neuro-inflammatory reflex and, consequently, to increased activity of the afferent vagal nerve. This activation might interfere with afferents of other compartments of the vagus nerve, originating from the mechanoreceptors of the heart. Chronic summation or interference of the combined activation might have changed the “prior” for starting and regulating the activation of the solitary nucleus and may therefore lead to reduced coupling with dorsal nucleus of the nervus vagus. Consequently, parasympathetic modulation of the heart rate decreases. Information about autoimmune activation is relayed to midbrain centres and initiates sickness behavior. *Behavior and conscious representation (right side):* Sickness behavior reduces locomotor activity, increases sleepiness and at the same time sleep disorder. Previous activations of sickness behavior might have changed the prior for triggering sickness behavior, and it becomes learned as being a chronic state. This leads to secondary deconditioning of the patients, loss of muscle strength and chronic changes in heart rate variability. Further processing of the signal induces the feeling of fatigue, loss of drive and anhedonia. The conscious processes start to interfere with focused attention, leading to enduring concentration deficits, which is fostered by deconditioning and chronic changes of heart rate variability

We would like to propose two different explanatory frameworks for fatigue and HRV changes in MS. First, by a strictly mechanical explanation, stimulation of the vagus nerve might act in a similar manner as stimulation of other neuronal structures. As has been shown in different contexts, such stimulation follows an inverted U-shaped curve: under- and overstimulation result in a similar dysfunction, whereas moderate levels of stimulation increase the effectivity of processing. If this assumption is correct, acute and chronic stimulation of the vagal nerve in autoimmune diseases might lead to a partial loss of its ability to regulate HRV (see Fig. 2, left side, vagal setpoint). Sander et al. [65] argue along the same lines. Patients with weak to moderate fatigue demonstrated expected changes in PANS-related HRV in response to a biofeedback intervention. Thus, weak to moderate levels of fatigue would belong to the upper part of the inverted U-shape. However, patients with severe fatigue did not demonstrate changes in PANS-related HRV in response to the biofeedback training. These patients would be localized on the right bow of the inverted U-shape. Second, Smith et al. [24] assume that adaption of the priors serving in the control of homoeostatic processing can take place because of a repetitive (over-)stimulation of the vagal afferents. Figure 2 offers an adapted version of our previous model 1 and at the same time an overview from previous models on the possible changes that may lead to chronic and self-stabilizing feelings of fatigue. This would not be a pure mechanical explanation for fatigue, it relies on learning processes at various biological, neuronal and conscious levels.

Although many of the results matched the predictions of our model, direct evidence from correlational studies between fatigue levels, HRV, and inflammatory processes is still weak. Most of the studies we reviewed focused on HRV in MS, and many were performed based on the assumption that HRV changes represent neural damage, which means that they did not consider HRV changes as a side aspect of a working neuro-inflammatory reflex. Therefore, they included patients with a different disease course and disease duration. Failing to differentiate MS course in the analyses may have led to discrepant data on sympathetic cardiovascular tone in MS. On the other hand, irrespective of disease course, RRMS, PPMS and variant-undefined MS patients were found to have lower PANS-related HRV than HC. This general finding argues that changes in PANS-related HRV do not depend on the existence of a lesion, brain atrophy or functional disability. The studies directly focusing on fatigue and HRV also displayed that the feeling of fatigue is related to changes in PANS-related HRV. This fits our hypothesis; however, the number of included patients was small, simultaneous measures of cytokines, HRV changes, and fatigue have not been done yet, and experimental variations in fatigue, HRV, or cytokine levels are lacking.

Convincing evidence for our assumption would depend on cross-correlational studies on fatigue, HRV and inflammation in MS and on longitudinal studies in RRMS patients. The prediction is that in newly diagnosed MS patients, PANS-related HRV would be lower than in HCs, and decreases even further shortly before a relapse. Methylprednisolone is effective in treating MS-related relapses and therefore an easily testable prediction is that this treatment would lead to an increase in PANS-related HRV. A similarly interesting question is, whether interventions focusing on biofeedback training could boost PANS-related HRV in MS patients, and if such an effect is associated with reductions in experienced fatigue. In this respect, it would also be interesting to examine whether longitudinal studies show such an adaptation and whether treatment can induce some recovery to prior environmental settings.

We explore the possibility that processing of chronic bodily inflammation perpetuates ANS changes in order to maintain allostasis [94], leading to different patterns of HRV activity, and that fatigue might be another, subjective, representation of such processing. The results of our review do not exclude this possibility, and assuming that our hypothesis turn out to be proven, that would open a new field of development of new interventions to treat MS-related fatigue.
